# Streptococcus Gordonii Empyema: A Case Report and Review of Empyema  

**DOI:** 10.7759/cureus.1159

**Published:** 2017-04-12

**Authors:** Amanda M Krantz, Felicia Ratnaraj, Manasa Velagapudi, Mridula Krishnan, Nagarjuna R Gujjula, Pamela A Foral, Laurel Preheim

**Affiliations:** 1 School of Medicine, Creighton University; 2 Internal Medicine, Creighton University Medical Center; 3 Internal Medicine, Creighton University School of Pharmacy and Health Professions

**Keywords:** streptococcus gordonii, empyema, splenic abscess

## Abstract

*Streptococcus gordonii *(*S. gordonii) *is a pioneer oral bacteria that is recognized as an agent of bacterial endocarditis. However, an extensive review of the literature revealed no reported case of* S. gordonii* causing empyema. We present a case of a 65-year-old male who presented with respiratory distress. Physical examination revealed several dental caries with decreased breath sounds in the bibasilar regions. A computed tomography (CT) scan of the chest and abdomen demonstrated left-sided pleural effusion and a 4.3 cm x 2.8 cm splenic abscess. He received intravenous (IV) antibiotics, and his blood cultures remained negative. Drainage of the splenic abscess grew *S. gordonii.* A CT-guided thoracentesis yielded 450 ml of exudative fluid. Pleural fluid cultures grew *S. gordonii*. A CT scan of the head and neck ruled out an intra-oral abscess. He received six weeks of IV penicillin with a follow-up CT scan showing resolution of both the splenic abscess and the left parapneumonic effusion.

## Introduction

Viridans streptococci, including *Streptococcus gordonii (S. gordonii)*, are pioneer oral bacteria often associated with dental plaque formation. *S. gordonii* is a gram-positive, non-motile cocci. It is a facultative anaerobe. *S. gordonii* has been well established as an inhabitant of the mouth and as an agent of endocarditis.

In the pre-antibiotic era, *S. pneumoniae*, *S. pyogenes,* and *S. aureus* were the most common pathogens associated with empyema. However, anaerobes have also been identified in a significant number of empyemas either as sole organisms or in mixed cultures.* S. gordonii *has not previously been reported as a cause of empyema. 

## Case presentation

A 65-year-old Caucasian male with a history of tobacco abuse, diabetes mellitus, and depression was admitted to a community hospital with hypotension, fever, and respiratory distress. Initial laboratory evaluations performed at the community hospital were within normal reference ranges and did not reveal leukocytosis. Chest radiographs revealed left middle lobe pneumonia so he was started on intravenous (IV) levofloxacin, 500 mg daily, with a transition to oral levofloxacin, 500 mg daily, for one week. He was transferred to a tertiary care center when his fever did not resolve and he developed new symptoms of abdominal pain. Informed consent was obtained upon transfer. 

**Figure 1:**
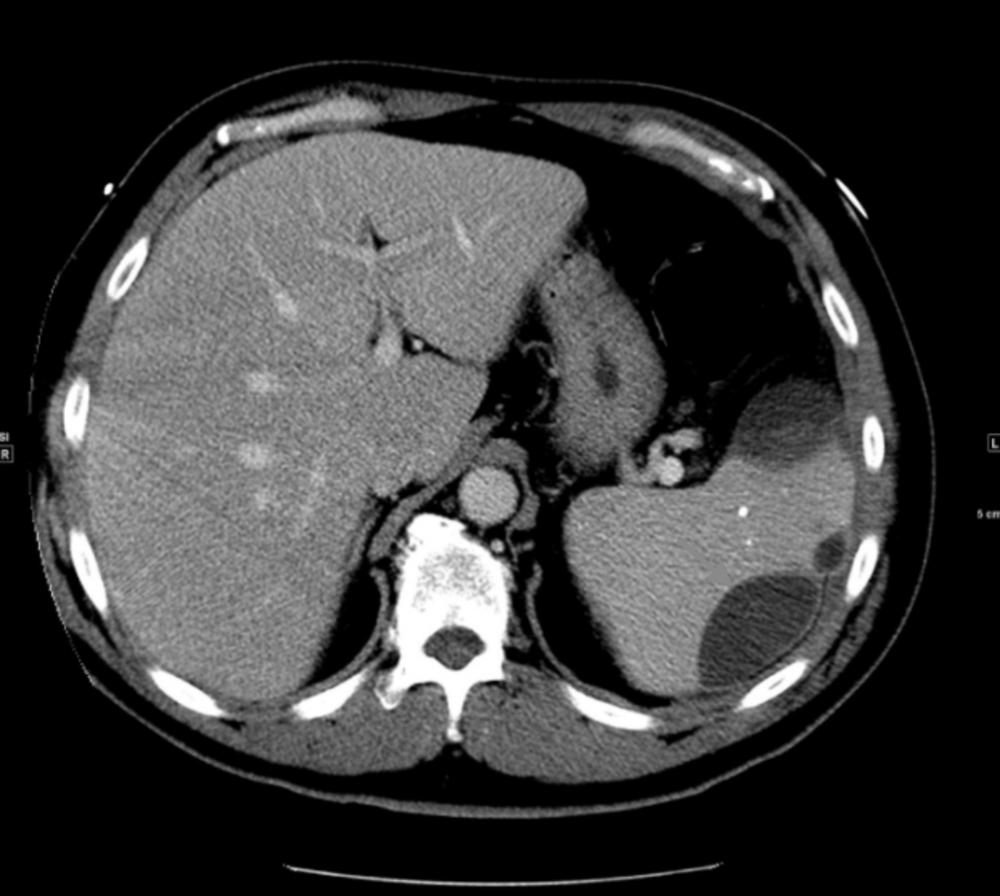
Splenic Abscess A computed tomography (CT) of the chest and abdomen demonstrated a splenic fluid collection measuring 4.3 cm x 2.8 cm, clinically suspicious for an abscess.

On presentation, he was in significant respiratory distress requiring intubation, mechanical ventilation, and vasopressors. Physical examination revealed several dental caries with missing teeth and a draining socket. Decreased breath sounds were noted in the bibasilar regions. A computed tomography (CT) of the chest and abdomen demonstrated left-sided pleural effusion and a splenic fluid collection measuring 4.3 cm x 2.8 cm was clinically suspicious for a splenic abscess (Figure 1).

He received weight-based dosing of IV vancomycin and piperacillin-tazobactam. Blood cultures were negative, procalcitonin was 6.51 ng/ml and lactic acid was 0.9 mmol/L. Drainage of the empyema was performed and cultures yielded a rare species, *S. gordonii*, susceptible to penicillin, vancomycin, ceftriaxone, clindamycin, erythromycin, and levofloxacin. The sensitivities were as follows: sensitivity to penicillin (minimum inhibitory concentration (MIC) 0.094), vancomycin (MIC 1), ceftriaxone (MIC 0.38), clindamycin (MIC 0.023), erythromycin (MIC 0.023), and levofloxacin (MIC 0.5). A CT-guided thoracentesis yielded 450 ml of exudative fluid with 4,329 nucleated white blood cells/mm 3 (79% neutrophils). The culture of the splenic abscess also grew *S. gordonii.* A CT scan of the head and neck, including maxillofacial views, was negative for odontogenic infection, neck abscess, or significant cervical lymphadenopathy. 

The patient received six weeks of IV penicillin, 20 million units daily. Follow-up CT scan after treatment showed resolution of both the splenic abscess and the left parapneumonic effusion. He improved clinically and tolerated the medication without any side effects. After discharge, the patient was maintained on 875 mg of amoxicillin twice daily for 10 months per recommendation from the infectious disease team. 

## Discussion

*S. gordonii *is a gram-positive, non-motile, cocci, facultative anaerobe member of the viridans group [1]. *S. gordonii* is commonly found in the mouth and is capable of spreading to extraoral sites, causing systemic infection [1]. *S. gordonii*, along with *S. sanguinis* and *S. oralis,* is commonly found in blood cultures of patients with infective endocarditis [2]. It is capable of colonizing platelet-fibrin thrombi on abnormal heart valves or the endocardium. It has also been reported in a case series of septic arthritis [3]. However, an extensive review of the literature revealed this to be the first case of *S. gordonii* empyema to be reported.

Empyema should be suspected in any new onset of pleural effusion in a patient who has had recent pneumonia, malignancy, or tuberculosis. An empyema can also follow a penetrating chest trauma or extension from a sub-diaphragmatic or paravertebral abscess [4]. Early diagnosis and complete drainage are critical as patients with empyema have higher rates of hospital admission, prolonged hospital stays, and increased mortality [5]. 

Radiographic imaging plays a key role in the evaluation and management of empyema [6]. Optimal evaluation of empyema requires a chest CT scan with contrast [7]. The criteria to consider thoracentesis is a thickened parietal pleura on a contrast-enhanced CT scan, a finding suggestive of empyema [6]. Based on the clinical presentation, it is difficult to ascertain whether the splenic abscess was secondary to the empyema or vice versa. The novelty of this case lies in it being the first known presentation of *S. gordonii *in an empyema. 

Fluid obtained by thoracentesis should undergo microbiologic analysis with appropriate stains and cultures. Samples of the fluid should also be sent for a cell count with differential and chemistries; pH should be determined with a blood gas analyzer [8]. Pleural fluid with a pH < 7.20 suggests drainage per the consensus guidelines from the American College of Chest Physicians [8]. Early surgical consultation is required because the majority of these patients will require thoracoscopic intervention or surgical debridement [9].  

Tube thoracostomy, video-assisted thoracoscopic surgery (VATS), open decortication, and open thoracostomy are the four drainage options for empyema. Tube thoracostomy is the most commonly preferred and the least invasive method of drainage of thoracic empyema [4, 10]. 

Complete and appropriate management of thoracic empyema includes systemic antibiotic therapy, adequate pleural fluid drainage, and obliteration of the empyema [10]. Empiric therapy should include agents effective against the oral and upper airway aerobic and anaerobic flora. The optimal duration of therapy often ranges from four to six weeks of IV antibiotics; some patients may require a longer antibiotic course. Adequate pleural fluid drainage is demonstrated by a minimal chest tube output and repeat imaging by a CT scan indicating the lack of any large residual loculations. 

## Conclusions

*S. gordonii *is a gram-positive, non-motile, facultative anaerobe member of the viridans group streptococci and is most commonly found in the mouth. This case represents the first known presentation of *S. gordonii *in an empyema. Our patient presented with unique complications of an *S. gordonii* infection, namely, an empyema and a splenic abscess. Empyema fluid is typically purulent, has a pH < 7.20, and contains bacteria identified on gram stain and/or cultures. Appropriate therapy of an empyema includes adequate drainage and antimicrobial therapy directed against the identified pathogen(s). 
